# Targeting equity in early childhood: resource allocation in Sweden’s Extended Home Visiting Program

**DOI:** 10.1186/s12939-026-02867-2

**Published:** 2026-04-27

**Authors:** Sergio Flores, Anna Fäldt, Erik Grönqvist, Filipa Sampaio, Anna Sarkadi

**Affiliations:** 1 Department of Public Health and Caring Sciences, Uppsala University, Box 564, Uppsala, 751 22 Sweden; 2 Department of Women’s and Children’s Health, Uppsala University, Uppsala, Sweden; 3 Department of Learning, Informatics, Management and Ethics, Karolinska Institute, Stockholm, Sweden; 4 Health Economics, Department of Medical Sciences, Uppsala University, Centre for Health Economic Research (HEFUU), Uppsala, Sweden

**Keywords:** Proportionate universalism, Health equity, Child health services, Resource allocation, Concentration index, Home visiting, Sweden

## Abstract

**Background:**

Sweden allocated SEK 354.5 million (approximately EUR 31.5 million) between 2018 and 2022 to extend home visiting services for families in socioeconomically disadvantaged areas. This initiative operationalises proportionate universalism within a decentralised welfare system where 21 regional governments hold authority over healthcare delivery. We examined whether program resources reached areas of greatest need and how targeting varied across regions and over time.

**Methods:**

We analysed Swedish administrative register data aggregated to 12,258 small statistical area-year observations across four implementing regions during each region’s post-rollout period. Program dosage was measured as probability-weighted exposure based on patient registration patterns between areas and child health centres. Socioeconomic need was measured using the Care Need Index (CNI). We estimated targeting associations using linear regressions with year fixed effects and standard errors clustered at the area level. We assessed equity using the Concentration Index and a proportional allocation benchmark, and evaluated within-area heterogeneity in household vulnerability.

**Results:**

Program resources were positively associated with area-level need (beta = 0.101, 95% CI: 0.091–0.111, *p* < 0.001). Where the program operated, targeting exceeded proportional benchmarks (proportionality ratio = 1.33) and the Concentration Index was positive (CI = 0.074), indicating pro-poor allocation. However, only 47% of high-need areas had any program presence, and regional variation was substantial: Stockholm’s targeting association was over three times stronger than Skåne’s (beta = 0.145 vs. 0.043). Within-area analysis revealed that more heterogeneous areas received slightly lower dosage, suggesting area-based allocation partially misses within-neighbourhood inequalities.

**Conclusions:**

Sweden directed resources toward higher-need areas, with targeting that exceeded proportional benchmarks where the program was implemented. The primary equity challenge was not targeting accuracy but coverage: more than half of high-need areas lacked any program presence. Achieving equitable outcomes through proportionate universalism requires both accurate allocation formulas and sufficient implementation capacity to reach all areas of need.

**Supplementary Information:**

The online version contains supplementary material available at 10.1186/s12939-026-02867-2.

## Background

The persistence of health inequalities in Nordic welfare states -- despite universal access to services and generous social protection -- has been characterised as the “Nordic paradox” [1, 2]. In Sweden, disparities in early childhood health track closely with parental income, educational attainment, and migration background [3–5]. While Sweden achieves near-universal coverage for routine childhood services (over 95% vaccination rates), lower household education and foreign-born parental status are associated with reduced engagement with preventive services [6, 7]. These persistent disparities reveal a limitation of traditional universal service models: providing identical services to all populations can inadvertently sustain inequality when more advantaged groups are better positioned to access and benefit from them.

### Proportionate universalism as a policy response

Proportionate universalism -- maintaining universal service provision while directing additional intensity to those with the greatest need -- has emerged as the leading policy framework for addressing this challenge [8]. However, translating this concept into practice presents considerable operational difficulties [9]. A central tension arises in decentralised welfare systems such as Sweden’s, where national policy can set al.location criteria and provide earmarked funding, but 21 regional governments hold constitutional authority over healthcare organisation and delivery [10]. Understanding how this governance structure shapes the translation of equity intent into local practice is essential for informing future interventions.

### The extended home visiting program

Between 2018 and 2022, the Swedish government funded an Extended Home Visiting Program (EHVP) through targeted state grants to enhance child health care accessibility for socioeconomically vulnerable populations. The program builds upon Sweden’s universal Child Health Care (CHC) system, which traditionally offers one to two home visits during a child’s first year. The extended intervention significantly intensifies this contact, offering up to six home visits over the child’s first 15 months, conducted by interprofessional teams comprising CHC nurses and parental advisors from social services [11].

The program model was originally developed in Rinkeby, a socioeconomically disadvantaged suburb in Stockholm with high concentrations of immigrant families [12]. Critically, the model maintains a universal offer within geographically targeted areas: all families in designated neighbourhoods are offered the extended visits, avoiding the stigmatisation associated with individual family selection while concentrating resources where aggregate need is highest. The Rinkeby pilot achieved 94% participation among offered families, demonstrating both high acceptability and potential scalability [11, 13].

The program is designed to address pathways to inequality by lowering access barriers, delivering tailored support, and strengthening families’ navigation of social services within their home environment. However, whether these mechanisms operate as intended depends substantially on how the program is implemented at the regional level.

### Regional variation in implementation

The national rollout faced considerable adaptation challenges. The distribution of state grants was guided by the Care Need Index (CNI), a composite deprivation measure used for needs-based resource allocation in Swedish primary healthcare [14]. However, regions varied substantially in how they operationalised these resources. Stockholm and Örebro utilised CNI thresholds to designate implementation areas. Västra Götaland relied on police-identified “vulnerable areas” (*utsatta områden*), introducing a potential urban bias by directing resources primarily toward segregated metropolitan suburbs. Skåne transitioned from an initial broad allocation approach toward more targeted, CNI-informed models over the funding period.

The national allocation formula itself evolved during the funding period. For 2018–2019, distribution was based on broad local needs assessments; in 2020, a revised formula strictly targeted grants according to demographic risk factors [14, 15]. This policy revision coincided with improvements in regional targeting, providing a natural experiment in the effects of centralised refinement on local implementation.

An important feature of the Swedish context is patient choice: families select which Child Health Centre to register with rather than being assigned based on residential location, though proximity remains a strong determinant of choice. This means that within any given area, children may attend different CHCs -- some implementing the extended program, others providing standard care. This choice-based system introduces measurement complexity for area-level analyses of program exposure.

### Research objectives

This study examines whether the EHVP’s resources were allocated in accordance with the principles of proportionate universalism. We address three research questions:

1. To what extent was program dosage associated with area-level socioeconomic need, and how did this association vary across implementing regions?
2. Did the allocation of resources meet explicit equity benchmarks, including proportional allocation relative to need and pro-poor concentration of services?
3. How did targeting evolve over time, and did within-area household vulnerability attenuate the equity gains achieved through area-based allocation?

## Methods

### Analytical framework

The analysis proceeded in four stages, each addressing a distinct dimension of targeting and equity:

- **Stage A -- National targeting association**: Estimated the overall relationship between area-level need and program dosage using regression analysis with explicit success benchmarks.
- **Stage B -- Regional heterogeneity**: Quantified how targeting associations varied across the four implementing regions and tested whether differences were statistically significant.
- **Stage C -- Equity assessment**: Evaluated allocation using the Concentration Index and a proportional allocation benchmark, replacing residual-based classification approaches that can produce mechanical artefacts.
- **Stage D -- Within-area equity**: Examined whether within-area heterogeneity in household vulnerability moderated the targeting relationship, testing for ecological fallacy concerns.

### Study design and data sources

We conducted a targeting analysis using Swedish administrative register data. Data were obtained from multiple national registers linked via personal identification numbers: the Total Population Register, the Longitudinal Integration Database for Health Insurance and Labour Market Studies (LISA), and healthcare utilisation records from the National Board of Health and Welfare (Socialstyrelsen).

The primary geographic unit was the DeSO (*Demografiska statistikområden*), a small-area geography designed by Statistics Sweden containing 700–3,000 residents each, enabling stable demographic tracking while maintaining statistical confidentiality [16].

### Sample definition

The analytical sample was restricted to each region’s post-rollout period, defined by empirically determined rollout start years (Stockholm: 2018; Skåne, Västra Götaland, and Örebro: 2019), corresponding approximately to the point at which 10% of each region’s DeSOs had non-zero program dosage. Based on empirical examination of the data:

- Stockholm: 2018 onward
- Skåne: 2019 onward
- Västra Götaland: 2019 onward
- Örebro: 2019 onward

This restriction focuses the analysis on the period of active implementation, when meaningful variation in program dosage existed across areas. Full-panel estimates including pre-rollout years are reported in Supplementary Table S1 as a sensitivity check. The resulting sample comprised 12,258 DeSO-year observations from 3,398 unique DeSOs.

### Measures

**Program dosage (outcome).** Individual-level participation data were unavailable. We therefore constructed a probability-weighted dosage score for each DeSO, representing the probability that a child aged 0–5 residing in that area attended a CHC funded to deliver the extended program. This was calculated from patient registration data mapping the proportion of children in each DeSO to specific CHCs. A dosage of 0 indicates no access to extended-program CHCs; a dosage of 1 indicates complete coverage by extended-program CHCs. Among DeSOs with any program presence during the post-rollout period, the mean dosage was 0.31 (31% of children registered at an extended-program CHC); unconditionally, 73% of post-rollout DeSO-years had zero dosage, reflecting the staged geographic rollout.

**Area-level need: the Care Need Index (CNI).** Socioeconomic need was measured using an adapted version of the Care Need Index (CNI), originally developed for needs-based resource allocation in Swedish primary healthcare [14]. The standard CNI comprises seven weighted demographic factors; we retained the four components applicable to families with young children -- single parenthood, low maternal education, foreign-born status, and unemployment -- excluding three components designed for the general primary care population (proportion of adults over 65, proportion of children under 5, and recent residential mobility). We computed a Relative CNI for each DeSO (DeSO CNI / national mean CNI). Values above 1.0 indicate need above the national average.

**Household Vulnerability Index (HVI).** To assess within-area equity (Stage D), we constructed a Household Vulnerability Index (HVI) for each family, summing five binary risk indicators: (1) low parental education (primary/lower secondary); (2) foreign-born status; (3) parental unemployment; (4) single-parent household; and (5) parental common mental health disorder (depression, anxiety, or stress-related conditions, identified from healthcare registers). We computed both the DeSO-level mean HVI and the within-DeSO standard deviation to capture heterogeneity.

**Additional covariates.** We also examined the proportion of foreign-born parents, the proportion unemployed, the proportion with low education, and the proportion of single-parent households as individual predictors of dosage allocation in supplementary models. In sensitivity analyses, we tested additional parental risk factors including household disposable income, maternal age at birth, and receipt of public benefits (sickness insurance, parental allowance).

### Stage A: National targeting

We estimated the association between area-level need and program dosage using the model:

$$\eqalign{ & Dosage_{it}{\rm{ }} = {\rm{ }}beta_{0}{\rm{ }} + {\rm{ }}beta_{1}{\rm{ }}*{\rm{ }}RelativeCNI_{it}{\rm{ }} \cr & \,\,\,\,\,\,\,\,\,\,\,\,\,\,\,\,\,\,\,\,\,\,\,\,\,\,\,\,\,\,\,\,\,\, + {\rm{ }}gamma_{t}{\rm{ }} + {\rm{ }}epsilon_{it} \cr}$$

  

where *i* indexes DeSOs and *t* indexes years. Year fixed effects (gamma_t) control for national temporal trends. Standard errors are clustered at the DeSO level to account for within-area serial correlation. We additionally tested a quadratic specification to assess non-linearity.

To provide explicit success benchmarks (rather than relying solely on statistical significance), we computed:

- **Proportionality ratio**: The ratio of the estimated beta to the beta that would be implied by perfectly proportional allocation (where a DeSO with CNI = 2.0 receives exactly twice the average dosage).
- **High-need coverage**: The percentage of DeSOs with CNI > = 1.0 (and higher thresholds) that had any program presence.
- **Equity gap ratio**: The ratio of mean dosage in the highest-need CNI quartile (Q4) to the lowest (Q1).

### Stage B: Regional heterogeneity

We ran stratified regressions by region and tested for equality of coefficients across regions using a Wald test from a pooled model with region-CNI interactions.

### Stage C: Equity assessment

We evaluated allocation equity using two approaches that avoid the mechanical artefacts inherent in OLS-residual-based classification (where residuals must sum to zero, mechanically classifying approximately half of areas as “under-served” regardless of actual allocation patterns).

**Concentration Index (CI).** We computed the health-sector-standard Concentration Index [17], which measures the extent to which program dosage is concentrated among higher- or lower-need areas. The CI ranges from − 1 to + 1: positive values indicate dosage is concentrated among high-need areas (pro-poor); negative values indicate concentration among low-need areas; zero indicates no systematic relationship with need. We computed CIs nationally and by region.

**Proportional allocation benchmark.** We defined a benchmark in which each DeSO receives dosage proportional to its relative need:

$$\eqalign{ & Benchmark{\rm{ }}dosage_{i }= \cr & \,\,\,\left( {RelativeCNI_{i}{\rm{ }}/{\rm{ }}mean{\rm{ }}CNI} \right)*mean{\rm{ }}dosage \cr}$$

Deviations from this benchmark (actual minus benchmark) identify areas that receive more or less than their proportional share. Unlike OLS residuals, benchmark residuals are not constrained to sum to zero, so the proportion of areas classified as “under-allocated” reflects genuine under-coverage rather than a mathematical artefact. We classified DeSO-years into four categories based on need level (CNI > = 1.0) and benchmark performance (positive or negative deviation).

### Stage D: Within-area equity

To test whether area-based targeting effectively reaches vulnerable households, we examined whether within-DeSO heterogeneity in household vulnerability moderated the CNI-dosage relationship. We estimated:

$$\begin{gathered} Dosage_{it}{\text{ }}={\text{ }}beta_{0}{\text{ }}+{\text{ }}beta_{1}{\text{ }}*{\text{ }}CNI_{it}{\text{ }}+ \hfill \\ {\text{ }}\,\,\,\,\,\,\,\,\,\,\,\,\,\,\,\,\,\,\,\,\,\,\,\,\,\,\,\,\,\,\,\,beta_{2}{\text{ }}*{\text{ }}HVI_{SD_{it}}{\text{ }}+{\text{ }} \hfill \\ \,\,\,\,\,\,\,\,\,\,\,\,\,\,\,\,\,\,\,\,\,\,\,\,\,\,\,\,\,\,\,\,\,\,\,\,\,beta_3{\text{ }}*{\text{ }}PctHighHVI_{it}{\text{ }}+{\text{ }} \hfill \\ \,\,\,\,\,\,\,\,\,\,\,\,\,\,\,\,\,\,\,\,\,\,\,\,\,\,\,\,\,\,\,\,gamma_t{\text{ }}+{\text{ }}epsilon_{it} \hfill \\ \end{gathered} $$

  

where HVI_SD is the within-DeSO standard deviation of the Household Vulnerability Index, and PctHighHVI is the proportion of households with HVI > = 2. A negative coefficient on HVI_SD would indicate that more heterogeneous areas -- where vulnerable and non-vulnerable households coexist -- receive lower dosage, suggesting that area-based allocation partially misses within-neighbourhood inequalities.

### Sensitivity analyses

We conducted the following robustness checks:

4. **Full-panel comparison**: Re-estimated all models on the complete 2012–2022 panel (including pre-rollout zeros) to assess the impact of sample restriction.
5. **Cross-sectional specification**: Estimated the post-rollout model without year fixed effects (pure cross-section) to confirm that results were not driven by within-area temporal variation.
6. **Population weighting**: Re-estimated models weighting by the number of households per DeSO to ensure results were not driven by small, atypical areas.
7. **Additional risk factors**: Tested household income, maternal age, and benefits receipt as additional targeting predictors.
8. **Poverty rate robustness**: Tested the proportion of low-income households as an alternative need measure alongside CNI.
9. **Non-linear specification**: Estimated a quadratic CNI model and computed the turning point at which the marginal association between need and dosage changes sign.
10. **Post-rollout threshold**: Re-estimated the main model under alternative definitions of the post-rollout period (0%, 5%, 15%, 20%, 25% DeSO coverage thresholds) to confirm that results were not sensitive to the 10% cutoff.
11. **Two-part model**: Because 73% of post-rollout DeSO-year observations had zero dosage, we decomposed the main OLS specification into an extensive margin (logit model for any program presence) and an intensive margin (OLS conditional on non-zero dosage) to verify that the targeting association operated through both the decision to place the program and the level of dosage within served areas.

### Statistical analysis

All models used ordinary least squares with year fixed effects and DeSO-clustered standard errors. Analyses were performed using the fixest package in R (version 4.4.2). All code is available from the corresponding author upon request.

### Complementary qualitative data

To contextualise quantitative findings, we draw on qualitative data previously collected from Central Child Health Services teams implementing the intervention [18]. These data, from semi-structured interviews with program managers, provide context regarding targeting decisions, investment strategies, and coordination challenges that shaped the statistical patterns observed.

### Ethical approval

This study was approved by the Swedish Ethical Review Authority (Etikprovningsmyndigheten, Dnr 2022-00783-01, approved 2022-03-08), with an amendment for additional register variables (Dnr 2025-01537-02, approved 2025-03-11). The study uses de-identified register data; informed consent was waived by the ethics authority. Qualitative data drawn upon for contextualisation were collected under the same ethical approval, with verbal informed consent obtained from all interview participants [18].

## Results

### Descriptive statistics

Table 1 presents sample characteristics for the post-rollout analytical sample. The 3,398 unique DeSOs exhibited substantial variation in socioeconomic need: Relative CNI ranged from 0 to 3.66, with a mean of 1.10 (SD = 0.81). Program dosage was highly skewed, with 73% of DeSO-years having zero exposure, reflecting staged implementation. Among DeSO-years with non-zero dosage, mean dosage was 0.31 (SD = 0.22), indicating that in areas where the program operated, approximately 31% of children aged 0–5 were registered at an extended-program CHC.

**Table 1:** Descriptive statistics for the post-rollout analytical sample (*N* = 12,258 DeSO-years; 3,398 unique DeSOs)

Variable	Overall	Stockholm	Skåne	Västra Götaland	Örebro
N (DeSO-years)	12,258	5,274	2,851	3,499	634
Program dosage, mean (SD)	0.083 (0.197)	0.085 (0.205)	0.155 (0.237)	0.028 (0.124)	0.051 (0.154)
Relative CNI, mean (SD)	1.10 (0.81)	1.14 (0.82)	1.16 (0.80)	1.00 (0.80)	0.94 (0.74)
Prop. foreign-born, mean (SD)	0.315 (0.245)	0.339 (0.251)	0.325 (0.240)	0.283 (0.239)	0.250 (0.216)
Prop. unemployed, mean (SD)	0.119 (0.165)	0.120 (0.156)	0.136 (0.187)	0.106 (0.159)	0.112 (0.165)
Prop. low education, mean (SD)	0.058 (0.117)	0.059 (0.119)	0.063 (0.118)	0.054 (0.116)	0.051 (0.106)
Prop. single parent, mean (SD)	0.072 (0.092)	0.080 (0.093)	0.066 (0.088)	0.066 (0.092)	0.071 (0.097)

Regional descriptive statistics revealed notable differences across implementing regions. Stockholm and Skåne had slightly higher mean CNI scores (1.14 and 1.16, respectively) than Västra Götaland (1.00) and Örebro (0.94). The proportion of foreign-born parents was highest in Stockholm (0.34) and Skåne (0.33), and lowest in Örebro (0.25). A comparison of implementing versus non-implementing regions showed that implementing regions had higher proportions of foreign-born parents (0.31 vs. 0.27), lower unemployment (0.17 vs. 0.26), and comparable CNI scores (1.06 vs. 0.98) (Supplementary Table S6).

### Stage A: National targeting

The EHVP demonstrated a significant positive association between area-level need and program dosage (beta = 0.101, SE = 0.005, 95% CI: 0.091–0.111, *p* < 0.001; Table 2). A one-unit increase in relative CNI -- equivalent to moving from the national average to twice the national average need -- was associated with a 10.1% point increase in program dosage.

**Table 2:** National targeting models: association between relative CNI and program dosage

	(1) Post-rollout, year FE	(2) Post-rollout, cross-section	(3) Full panel, year FE
Relative CNI	0.101***	0.103***	0.076***
	(0.005)	(0.005)	(0.004)
95% CI	[0.091, 0.111]	[0.093, 0.113]	[0.068, 0.084]
N	12,258	12,258	18,900
Adjusted R-squared	0.190	0.176	0.194

Standard errors clustered at DeSO level in parentheses. *** *p* < 0.001

The targeting association was robust to specification. Cross-sectional estimation without year fixed effects yielded a virtually identical coefficient (beta = 0.103, SE = 0.005), confirming that the result reflects cross-area variation in need and dosage rather than within-area temporal dynamics. The full-panel estimate including pre-rollout years was 25% smaller (beta = 0.076, SE = 0.004), demonstrating the diluting effect of pre-rollout zeros.

A quadratic specification revealed significant non-linearity (Wald test: chi-squared = 76.9, *p* < 2.2 × 10^-16), with the quadratic CNI term positive and significant. The estimated turning point -- below which the marginal association is negative and above which it is positive -- occurred at relative CNI = 0.49. 77% of post-rollout observations exceeded this turning point, and all areas classified as high-need (CNI > = 1.0) fell within the region of positive, increasing targeting.

### Explicit success benchmarks

To move beyond statistical significance toward substantive interpretation, we evaluated targeting against three explicit benchmarks (Table 3).

**Table 3:** Targeting scorecard: explicit success benchmarks (post-rollout, implementing regions)

Benchmark	Value	Interpretation
Proportionality ratio (actual / proportional beta)	1.33	133% of perfect proportionality
High-need coverage (CNI > = 1.0)	46.7%	Proportion of high-need areas with any dosage
High-need coverage (CNI > = 1.5)	60.6%	Proportion of very-high-need areas reached
High-need coverage (CNI > = 2.0)	71.8%	Proportion of extreme-need areas reached
Equity gap ratio (Q4/Q1 mean dosage)	5.38	Highest-need quartile receives 5.4x lowest
Concentration Index	0.074	Positive = dosage favours higher-need areas

The proportionality ratio of 1.33 indicates that the observed targeting association was 133% of what perfectly proportional allocation would require -- that is, where the program operated, resources were directed to high-need areas at a rate exceeding strict proportionality. The equity gap ratio of 5.38 further confirms substantial differentiation: DeSOs in the highest-need quartile received on average 5.38 times the dosage of those in the lowest quartile (Supplementary Table S5).

However, high-need coverage was incomplete. Only 46.7% of DeSOs with CNI > = 1.0 had any program presence during the post-rollout period. Coverage improved at higher need thresholds -- 60.6% for CNI > = 1.5 and 71.8% for CNI > = 2.0 -- suggesting that implementation prioritised the most disadvantaged areas, but substantial coverage gaps persisted.

### Stage B: Regional heterogeneity

**Fig. 1:**
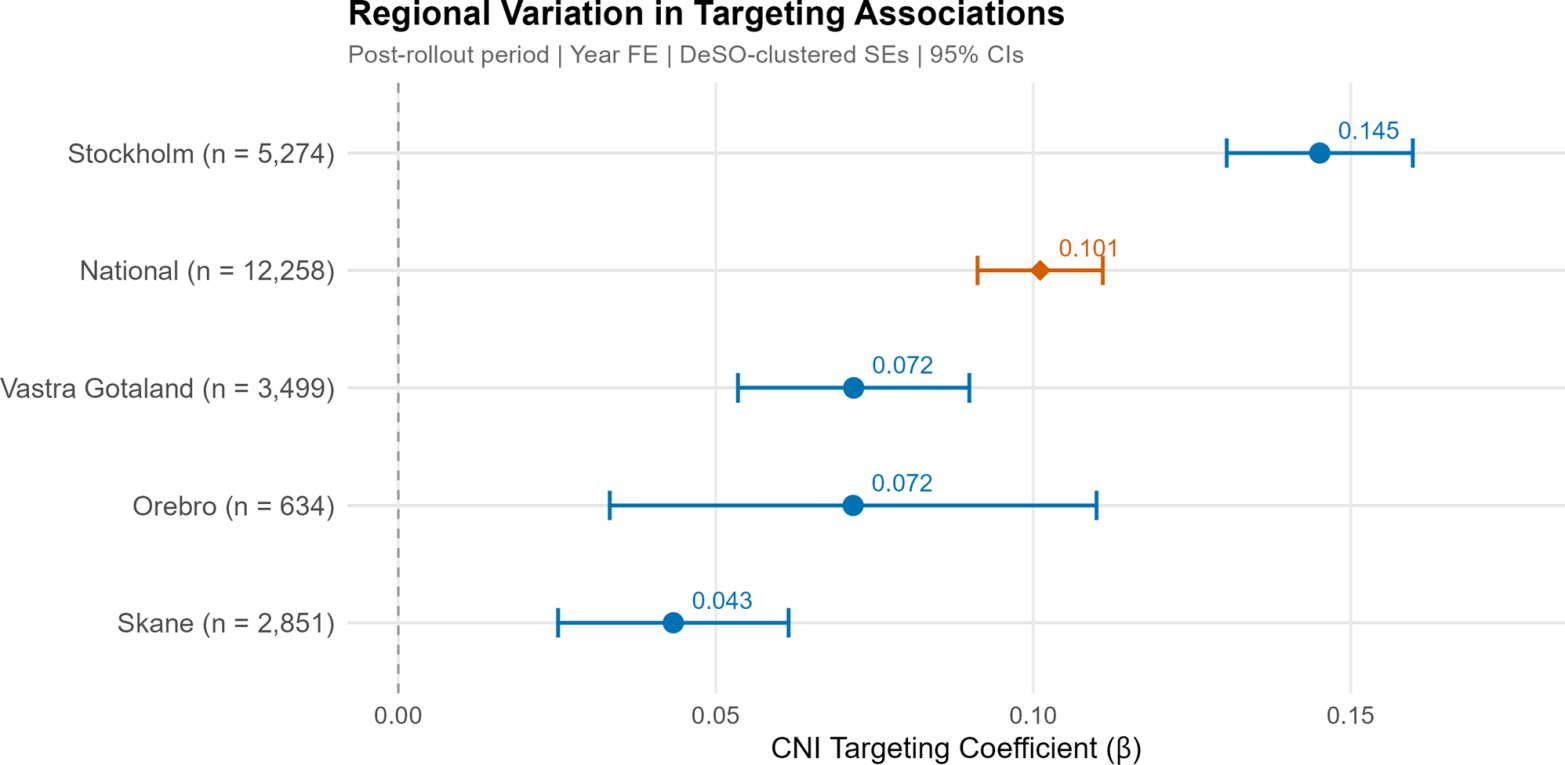
Regional variation in targeting associations. Post-rollout period, year fixed effects, DeSO-clustered standard errors, 95% confidence intervals

Regional targeting associations varied substantially (Fig. 1). Stockholm exhibited the strongest association (beta = 0.145), followed by Västra Götaland (beta = 0.072), Örebro (beta = 0.072), and Skåne (beta = 0.043). A formal Wald test confirmed that these coefficients were statistically distinguishable (chi-squared = 27.6, *p* < 2.2 × 10^-16). Stockholm’s targeting association was more than three times stronger than Skåne’s, and all pairwise differences from Stockholm were statistically significant (*p* < 0.001).

Regional Concentration Indices mirrored this pattern: Stockholm CI = 0.102, Örebro CI = 0.060, Västra Götaland CI = 0.046, and Skåne CI = 0.029 (Supplementary Table S3). All regional CIs were positive, indicating pro-poor allocation in every implementing region, though the magnitude varied considerably.

### Stage C: Equity assessment

**Fig. 2:**
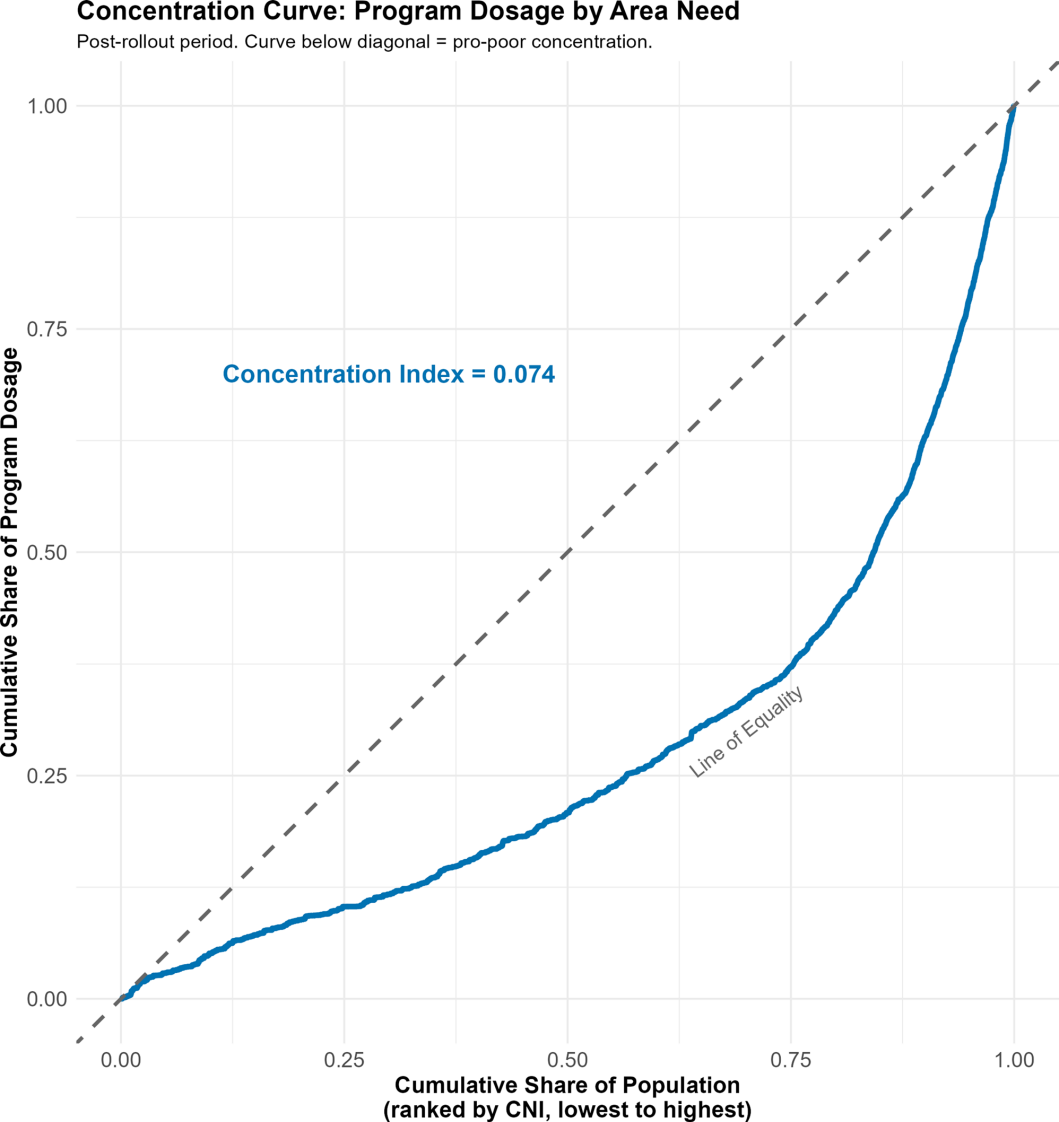
Concentration curve: cumulative population share (ranked by ascending CNI) versus cumulative program dosage share. Post-rollout period, implementing regions

The national Concentration Index for the post-rollout period was 0.074 (Fig. 2), indicating that program dosage was concentrated among higher-need areas. The concentration curve lay consistently below the line of equality, confirming that the cumulative share of dosage exceeded the cumulative share of population when ranked by ascending need.

**Fig. 3:**
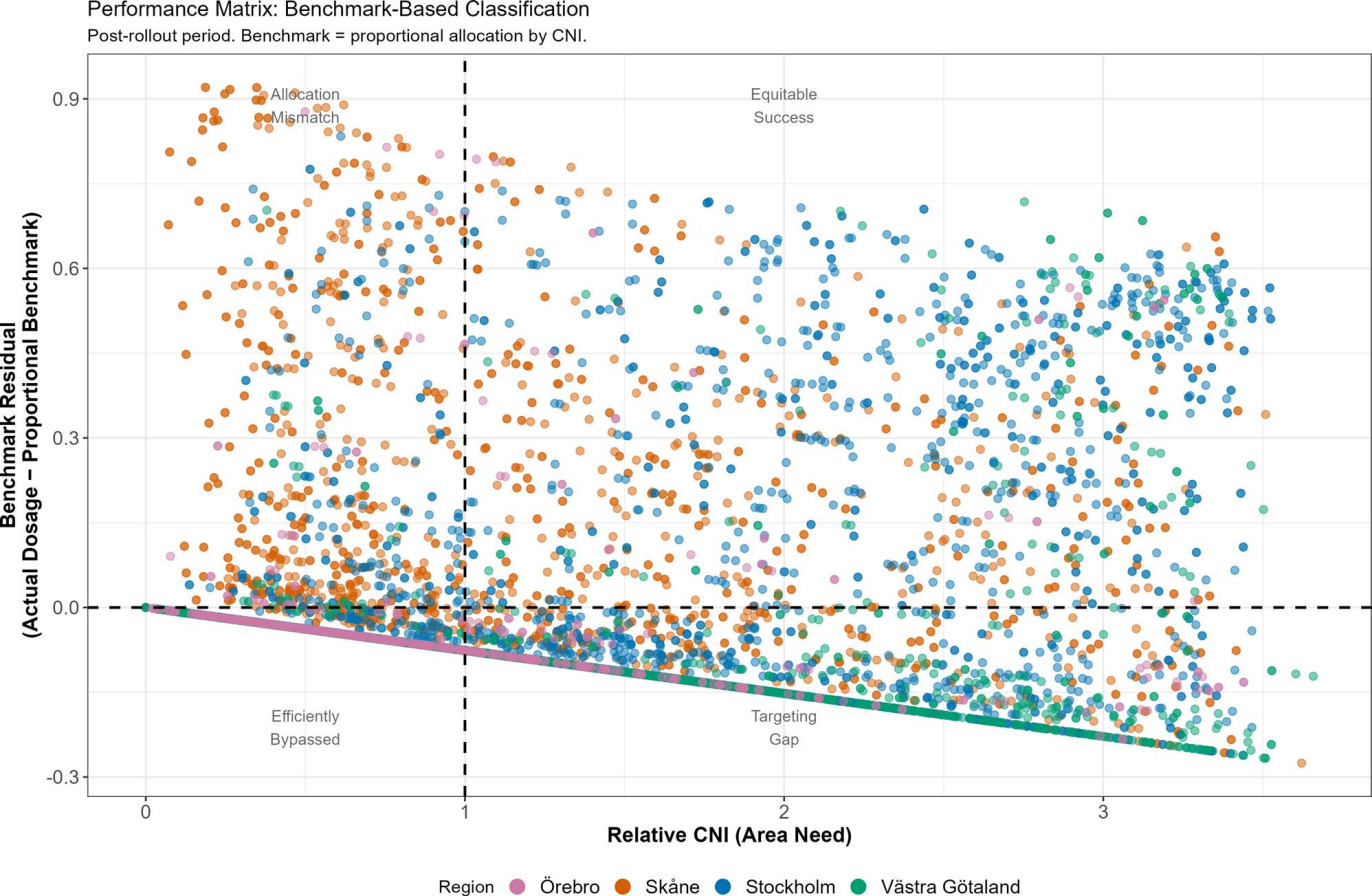
Benchmark-based performance matrix. DeSOs classified by area need (relative CNI) and benchmark residual (actual dosage minus proportional benchmark). Post-rollout period, implementing regions

The proportional allocation benchmark analysis classified DeSO-years based on whether they received more or less dosage than their need level would warrant under strict proportionality (Fig. 3). Under this benchmark:

- 10.7% of DeSO-years received dosage exceeding their proportional share (positive benchmark residuals).
- 82.5% received less than their proportional share (negative benchmark residuals).
- Among high-need areas (CNI > = 1.0): 27.2% achieved “equitable success” (received above-proportional dosage); 72.8% had “targeting gaps” (received below-proportional dosage).

This distribution -- with 82.5% of areas receiving below-proportional dosage -- reflects the genuine incomplete coverage of the program rather than a mechanical artefact. When compared with the conventional OLS-residual approach (which by construction classifies approximately 50% as under-allocated), the benchmark approach reveals that the original quadrant analysis substantially underestimated the true extent of coverage gaps (Supplementary Table S4).

### Stage D: Within-area equity

Area-level CNI and aggregated HVI were highly correlated (*r* = 0.86), confirming that vulnerable households cluster in high-need areas. However, within-DeSO HVI heterogeneity was substantial: the mean within-area standard deviation of HVI was 0.80 (median = 0.82, IQR: 0.65–0.97), indicating meaningful variation in household vulnerability even within small statistical areas.

Models testing the moderating role of within-area heterogeneity revealed a significant negative association between HVI standard deviation and program dosage (beta = -0.240, SE = 0.028, *p* < 0.001; Table 4, Model 2). Areas with greater heterogeneity in household vulnerability -- where high- and low-vulnerability families coexist -- tended to receive lower program dosage, even after controlling for mean area-level need.

**Table 4:** Within-area heterogeneity models: CNI targeting coefficient with HVI heterogeneity controls

	(1) Base	(2) Additive	(3) Full (revised)	(4) With prop. foreign-born
Relative CNI	0.101***	0.107***	0.136***	0.150***
	(0.005)	(0.005)	(0.015)	(0.021)
HVI_SD		-0.240***	-0.157***	-0.162***
		(0.028)	(0.031)	(0.031)
% High HVI		0.106***	0.297***	0.327***
		(0.022)	(0.071)	(0.079)
Prop. unemployed			-0.080	-0.118
			(0.052)	(0.062)
CNI x HVI_SD			-0.080*	-0.078*
			(0.034)	(0.034)
Prop. foreign-born				-0.053
				(0.052)
VIF for CNI	1.0	1.7	12.3	52.2
Adjusted R-squared	0.190	0.208	0.209	0.209
N	12,258	12,258	12,258	12,258

All models include year fixed effects. Standard errors clustered at DeSO level in parentheses. *** *p* < 0.001, * *p* < 0.05. Model 4 is reported for transparency but suffers from severe multicollinearity: the proportion of foreign-born residents is a direct component of the CNI composite score (*r* = 0.98, VIF > 50). Model 3 excludes this variable. The proportion unemployed is retained (*r* = 0.33 with CNI)

**Fig. 4:**
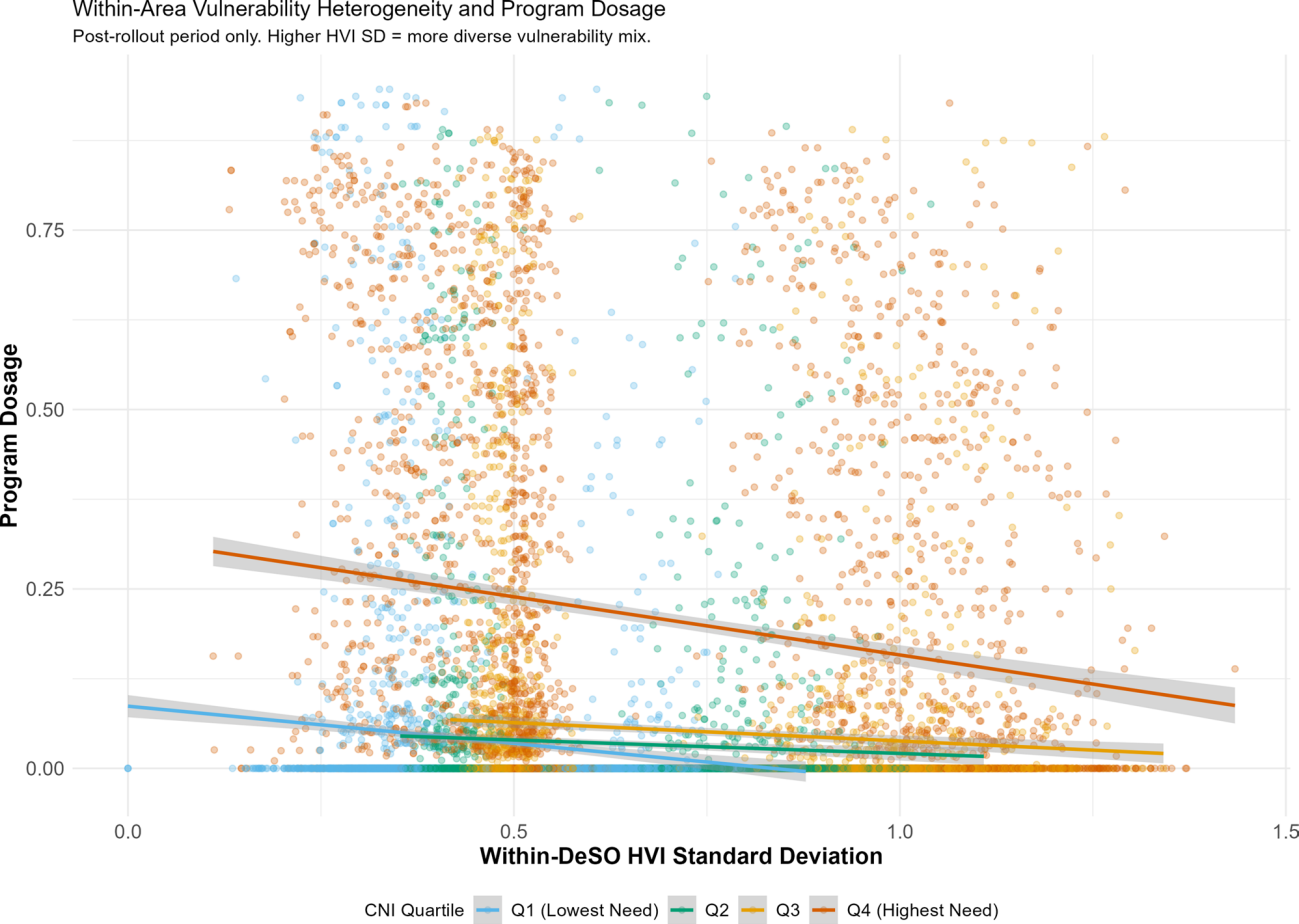
Within-DeSO HVI heterogeneity and program dosage by CNI quartile. Post-rollout period, implementing regions

The CNI targeting coefficient remained robust and strengthened slightly when controlling for within-area heterogeneity (beta = 0.107 in the additive model with HVI_SD and percent high-vulnerability, compared to 0.101 in the base model; Table 4). The coefficient increased further to 0.136 in the model with the CNI-by-HVI_SD interaction term and the proportion unemployed as an additional control. This suggests that area-based targeting is effective at directing resources to high-need neighbourhoods, but the ecological fallacy concern raised by within-area heterogeneity represents a genuine, though quantitatively modest, limitation of the area-level allocation approach (Fig. 4).

### Temporal evolution

Year-specific cross-sectional models estimated on the post-rollout sample revealed variation in targeting over time (Table 5). The CNI coefficient was already substantial in 2018 (beta = 0.101, the first year of Stockholm’s rollout), dipped in 2019 (beta = 0.078) as the three other regions entered at an earlier stage of implementation, and then strengthened to 0.117 in 2020, coinciding with the national revision of the grant distribution formula. The coefficient stabilised around 0.10 in 2021–2022.

**Table 5:** Temporal evolution of targeting coefficients (post-rollout, year-by-year cross-sectional models)

Year	*N*	CNI coefficient	SE	95% CI
2018	738	0.101	0.011	[0.079, 0.122]
2019	2,319	0.078	0.007	[0.065, 0.091]
2020	2,759	0.117	0.007	[0.103, 0.130]
2021	3,243	0.099	0.005	[0.089, 0.110]
2022	3,199	0.106	0.005	[0.095, 0.116]

DeSO-clustered standard errors. All coefficients significant at *p* < 0.001

### Sensitivity analyses

Results were robust across all sensitivity checks:

- **Population weighting** changed the national coefficient by less than 5% (beta = 0.106 weighted vs. 0.101 unweighted).
- **Additional parental risk factors** (household income, maternal age, benefits receipt) did not substantively alter the CNI coefficient. The proportion of foreign-born parents was the strongest individual predictor (beta > 0.30), consistent with the program’s origins in an area with high immigrant concentration.
- **Poverty rate robustness**: CNI dominated the proportion of low-income households in joint models; the poverty measure was not significant after controlling for CNI.
- **Full-panel results**: The full-panel CNI coefficient (beta = 0.076) was 25% smaller than the post-rollout estimate, confirming that pre-rollout zeros dilute but do not eliminate the targeting signal.
- **Post-rollout threshold**: The 10% DeSO coverage threshold used to define the post-rollout period was tested against alternative cutoffs (0%, 5%, 15%, 20%, 25%). The CNI coefficient ranged from 0.076 (no restriction) to 0.115 (15% threshold) and was significant at *p* < 0.001 under all specifications (Supplementary Table S7).
- **Two-part model**: Because 73% of post-rollout observations had zero dosage, we decomposed the main OLS result using a two-part specification. The extensive margin (logit) confirmed that higher-need areas were significantly more likely to have any program presence (odds ratio = 3.83, 95% CI: 3.47 to 4.23). The intensive margin (OLS conditional on non-zero dosage, *n* = 3,298) confirmed that among areas with the program, higher need predicted greater dosage (beta = 0.058, 95% CI: 0.041 to 0.075). Both margins were significant at *p* < 0.001, supporting the pooled OLS specification. When demographic covariates were added, the CNI coefficient on the extensive margin became non-significant while the proportion of foreign-born residents emerged as the dominant predictor of program placement, consistent with the program’s origins in areas with high immigrant concentration. The intensive margin coefficient for CNI remained significant in the expanded model (Supplementary Table S8).

## Discussion

This analysis provides empirical evidence on the implementation of proportionate universalism in a decentralised welfare state. The findings reveal a nuanced picture: Sweden’s Extended Home Visiting Program achieved pro-poor targeting that exceeded proportional benchmarks where the program operated, but coverage gaps left more than half of high-need areas without any program presence.

### Targeting accuracy versus coverage

The central finding -- a positive association between area-level need and program dosage (beta = 0.101) with a proportionality ratio of 1.33 -- indicates that the allocation formula and regional implementation translated equity intent into measurable resource differentiation. The Concentration Index of 0.074 confirms that dosage was concentrated among higher-need areas, constituting a positive departure from the inverse care law [19].

However, the magnitude of this association requires careful contextualisation. 73% of post-rollout DeSO-years had zero program dosage, reflecting the reality that the EHVP was a time-limited initiative (2018–2022) with insufficient resources to reach all areas of need simultaneously. Among DeSO-years with non-zero dosage, the mean was 0.31 -- indicating that in served areas, nearly one-third of children aged 0–5 were registered at an extended-program CHC. The primary equity challenge was therefore not the accuracy of targeting (which exceeded proportional benchmarks) but the breadth of coverage (only 47% of high-need areas reached).

This distinction between targeting accuracy and coverage has practical significance for policy evaluation. Judging a program solely by its overall targeting coefficient -- which pools served and unserved areas -- risks conflating two separate policy questions: “Does the program go to the right places?” and “Does the program go to enough places?” Our benchmarking approach allows these questions to be addressed separately.

### Regional variation

The substantial variation in regional targeting associations -- from Stockholm (beta = 0.145) to Skåne (beta = 0.043) -- reflects differences in implementation approach, regional context, and historical service development. We interpret these differences cautiously, noting several possible contributing factors.

Stockholm’s stronger targeting likely reflects both the direct influence of the Rinkeby pilot model and the region’s earlier adoption and longer experience with CNI-based allocation. Skåne’s weaker targeting may partly reflect its transition from a broad application-based model to more targeted allocation during the study period. Västra Götaland’s reliance on police-identified “vulnerable areas” rather than the CNI may have introduced an urban bias, directing resources toward segregated metropolitan suburbs while potentially bypassing socioeconomic disadvantage in the region’s extensive rural areas.

The observation that all regions achieved positive Concentration Indices -- ranging from 0.029 (Skåne) to 0.102 (Stockholm) -- suggests that pro-poor targeting was achieved regardless of the specific targeting criteria employed, though its intensity varied considerably. Whether these regional differences reflect administrative capacity, political priorities, geographic characteristics, or the specific targeting criteria used cannot be disentangled with the available data.

### Temporal dynamics and policy learning

The temporal pattern of targeting coefficients -- with a dip in 2019 when three new regions entered implementation, followed by strengthening to 0.117 in 2020 -- suggests a process combining top-down policy refinement with bottom-up organisational learning. The increase observed between 2019 and 2020 aligns with the national government’s revision of the grant distribution formula to better reflect local demographic risk factors [14, 15]. Previous qualitative research has documented how this national directive enabled regions to operationalise targeting more effectively by legitimising the focus on disadvantage and necessitating improved coordination between health and social services [18]. The COVID-19 pandemic, which overlapped with the later years of the study period, may have further shaped these dynamics by disrupting routine service delivery and disproportionately affecting disadvantaged communities [20].

This temporal pattern has implications for the evaluation of complex social interventions more broadly. Early-phase targeting coefficients may substantially underestimate a program’s long-run equity performance, suggesting that evaluation designs should account for implementation learning curves rather than treating targeting as static.

### Within-area equity and the ecological fallacy

The finding that within-DeSO HVI heterogeneity is negatively associated with program dosage (beta = -0.240) confirms that area-based targeting does not fully translate into household-level equity. Areas where vulnerable and non-vulnerable households coexist tend to receive slightly lower dosage than more homogeneously disadvantaged areas, even after controlling for mean area-level need.

This result must be interpreted in the context of Sweden’s patient choice system, in which families select their CHC rather than being assigned by residential location. Within any given DeSO, children may attend different CHCs -- some implementing the extended program, others providing standard care. The most vulnerable households may be less likely to exercise choice toward extended-program CHCs, or may cluster in micro-locations within the DeSO that are less proximate to program-implementing centres.

These findings underscore a fundamental tension in the program’s design. The universal offer within targeted areas was explicitly chosen to avoid the stigmatisation of individual family selection [11]. While this approach successfully achieved area-level pro-poor targeting, it appears insufficient to ensure household-level equity. Complementary strategies -- such as active outreach to the most vulnerable households within targeted areas, trust-building pre-visits, or tighter integration with antenatal care -- may be necessary to bridge this gap.

### Limitations

Several limitations should be acknowledged.

First, the dosage variable represents probability-weighted potential exposure based on patient registration patterns, not individual-level program participation. This introduces measurement error and means our estimates capture service availability rather than actual receipt. Given the within-area findings suggesting lower engagement by the most vulnerable households, area-level estimates may overstate the actual exposure of high-need families.

Second, our analysis cannot establish causal relationships between need and allocation. The observed associations may be influenced by unmeasured confounders, including regional political priorities, historical service infrastructure, and the geographic concentration of immigrant populations. We use associational language throughout to reflect this limitation.

Third, within-area heterogeneity analysis is constrained by the area-level design. While we computed within-DeSO HVI standard deviations and found them to moderate targeting, we cannot observe individual-level program participation. The ecological fallacy concern is partially but not fully addressed.

Fourth, the study focuses on resource allocation (inputs) rather than child health outcomes. Whether the observed targeting patterns translate into reduced health inequalities cannot be assessed with these data.

Fifth, residential mobility may introduce measurement error in the area-level dosage variable. Families who move between DeSOs during the year could be assigned to a different area than where they received or were eligible for the program. Three features mitigate this concern. DeSOs are small statistical areas (median 183 households with children in our sample), and many residential moves occur between areas with similar sociodemographic profiles, particularly within-municipality moves, which are the most common type in Sweden. The Swedish population register, which provides the denominators for our dosage variable, is updated annually, so the area-level aggregation reflects the year-end residential location. Our outcome variable captures area-level program availability rather than individual uptake, so the relevant question is whether the program was available in the area of residence, not whether a specific family used it. Nevertheless, residential mobility adds noise to the area-level estimates, reducing precision and, to the extent that it induces misclassification of area-level exposure, may attenuate the observed targeting coefficients toward zero.

Sixth, the analysis covers only the four regions that adopted the full extended home visiting model. These regions may represent a best-case scenario regarding motivation and leadership; equity performance could be lower in regions that implemented less intensive versions or no program at all.

Finally, length of time in Sweden -- a potentially important factor for foreign-born families’ engagement with health services -- was unavailable in our data and represents a gap in our analysis of risk factor targeting.

## Conclusions

Sweden’s Extended Home Visiting Program achieved pro-poor resource allocation that exceeded proportional benchmarks. Where the program operated, targeting was accurate and improving over time: the Concentration Index was positive across all implementing regions, and the proportionality ratio of 1.33 indicates that high-need areas received more than their proportional share of resources.

However, the primary equity challenge was coverage rather than accuracy. Only 47% of high-need areas had any program presence, and the within-area analysis revealed that area-based allocation partially misses household-level inequalities. The program’s approach of offering universal services within targeted geographic areas -- while effective at avoiding stigmatisation -- was insufficient to ensure that the most vulnerable families within those areas were reached.

These findings offer three lessons for the implementation of proportionate universalism. First, sufficient resources and implementation capacity must accompany allocation formulas; accurate targeting is of limited value if the program cannot reach enough areas. Second, centralised policy refinement can catalyse improvements in local targeting, as demonstrated by the step-change following Sweden’s 2020 formula revision. Third, area-based targeting, while a necessary foundation, should be complemented by individual-level engagement strategies designed to reach the most vulnerable families within targeted neighbourhoods.

## Electronic Supplementary Material

Below is the link to the electronic supplementary material.

Supplementary Material 1

## Data Availability

The data that support the findings of this study are available from Statistics Sweden and the National Board of Health and Welfare. Restrictions apply to the availability of these data, which were used under licence for the current study, and so are not publicly available. Data are available from the authors upon reasonable request and with permission of Statistics Sweden and the National Board of Health and Welfare, subject to ethical approval and secrecy assessment.

## Abbreviations

CHC: Child health centre
CI: Concentration index
CNI: Care need index
DeSO: Demografiska statistikområden (demographic statistical areas)
EHVP: Extended home visiting program
HVI: Household vulnerability index
LISA: Longitudinal integration database for health insurance and labour market studies
OLS: Ordinary least squares
SD: Standard deviation
SE: Standard error
SEK: Swedish Krona
