# Supplementary material for: Targeting equity in early childhood: resource allocation in Sweden’s Extended Home Visiting Program
Source: Int J Equity Health. 2026 Apr 27;25:112. doi: 10.1186/s12939-026-02867-2 (PMC13123165; doi:10.1186/s12939-026-02867-2)

# Supplementary Material

*Targeting Equity in Early Childhood: Resource Allocation in Sweden’s Extended Home Visiting Program*

Flores S, Fäldt A, Grönqvist E, Sampaio F, Sarkadi A

## Supplementary Tables

**Table S1. Comparison of Full Panel vs. Post-Rollout Targeting Estimates**

| **Model** | **N** | **CNI coefficient** | **SE** | **p-value** | **Adj. R²** |
| --- | --- | --- | --- | --- | --- |
| Full Panel (Year FE) | 18,900 | 0.0755 | 0.0041 | < 0.001 | 0.1939 |
| Post-Rollout (Year FE) | 12,258 | 0.1011 | 0.0050 | < 0.001 | 0.1895 |
| Post-Rollout (Cross-Section) | 12,258 | 0.1025 | 0.0050 | < 0.001 | 0.1756 |

*Note: Three models estimated: (1) full panel 2012-2022 with year FE, (2) post-rollout restricted sample with year FE, (3) post-rollout cross-sectional without year FE. Post-rollout restriction increases the CNI coefficient by approximately 34%, confirming that pre-rollout zeros dilute the targeting estimate.*

**Table S2. Within-Area Heterogeneity Models: CNI Targeting Coefficient with HVI Heterogeneity Controls**

| **Model** | **N** | **CNI coefficient** | **SE** |
| --- | --- | --- | --- |
| (1) Base (CNI only) | 12,258 | 0.101 | 0.005 |
| (2) Additive (CNI + HVI SD + % high-vulnerability) | 12,258 | 0.107 | 0.005 |
| (3) Full (revised, excl. prop. foreign-born) | 12,258 | 0.136 | 0.015 |
| (4) Expanded (incl. prop. foreign-born; for transparency) | 12,258 | 0.150 | 0.021 |

*Note: Four models testing whether within-area household vulnerability heterogeneity moderates the CNI-dosage targeting association. All models include year fixed effects and DeSO-clustered standard errors. Model 4 suffers from severe multicollinearity (VIF for CNI = 52.2) because proportion foreign-born is a component of the CNI composite (r = 0.98); it is retained for transparency only. See main text Table 4 for full coefficient details.*

**Table S3. Concentration Indices by Scope and Region**

| **Scope** | **Concentration Index** |
| --- | --- |
| National (all years) | 0.0526 |
| National (post-rollout) | 0.0737 |
| Stockholm (all years) | 0.0810 |
| Skåne (all years) | 0.0195 |
| Västra Götaland (all years) | 0.0295 |
| Örebro (all years) | 0.0365 |
| Stockholm (post-rollout) | 0.1021 |
| Skåne (post-rollout) | 0.0287 |
| Västra Götaland (post-rollout) | 0.0463 |
| Örebro (post-rollout) | 0.0595 |

*Note: Concentration Index (CI) computed using fractional rank method. CI > 0 indicates dosage concentrated among higher-need areas (pro-poor). All values are positive, confirming pro-poor allocation across all regions.*

**Table S4. Cross-Tabulation of OLS-Residual vs. Benchmark-Based Quadrant Classification**

| **OLS** | **Benchmark** | **N** |
| --- | --- | --- |
| 1. Equitable Success | 1. Equitable Success | 1,236 |
| 2. Targeting Gap | 1. Equitable Success | 82 |
| 3. Allocation Mismatch | 1. Equitable Success | 0 |
| 4. Efficiently Bypassed | 1. Equitable Success | 0 |
| 1. Equitable Success | 2. Targeting Gap | 60 |
| 2. Targeting Gap | 2. Targeting Gap | 3,464 |
| 3. Allocation Mismatch | 2. Targeting Gap | 0 |
| 4. Efficiently Bypassed | 2. Targeting Gap | 0 |
| 1. Equitable Success | 3. Allocation Mismatch | 0 |
| 2. Targeting Gap | 3. Allocation Mismatch | 0 |
| 3. Allocation Mismatch | 3. Allocation Mismatch | 795 |
| 4. Efficiently Bypassed | 3. Allocation Mismatch | 35 |
| 1. Equitable Success | 4. Efficiently Bypassed | 0 |
| 2. Targeting Gap | 4. Efficiently Bypassed | 0 |
| 3. Allocation Mismatch | 4. Efficiently Bypassed | 1,424 |
| 4. Efficiently Bypassed | 4. Efficiently Bypassed | 5,162 |

*Note: Compares the original OLS-residual quadrant approach (which mechanically forces ~50% negative residuals) with the proportional allocation benchmark approach (which does not). Under the benchmark, 82.5% of areas receive below-proportional dosage, reflecting genuine coverage gaps.*

**Table S5. Targeting Scorecard with Explicit Success Benchmarks**

| **Benchmark** | **National** | **Interpretation** |
| --- | --- | --- |
| A. Proportionality Ratio (actual beta / proportional beta) | 1.33 | 132.9% of perfect proportionality |
| B. High-Need Coverage (% of CNI>=1.0 with any dosage) | 46.7% | % of high-need areas reached |
| B. High-Need Coverage (% of CNI>=1.5 with any dosage) | 60.6% | % of very-high-need areas reached |
| B. High-Need Coverage (% of CNI>=2.0 with any dosage) | 71.8% | % of extreme-need areas reached |
| C. Equity Gap Ratio (Q4/Q1 mean dosage) | 5.38 | > 1 = pro-poor |
| D. Concentration Index (post-rollout) | 0.074 | > 0 = dosage favours high-need areas |

*Note: Three benchmarks: (A) Proportionality Ratio = actual targeting beta / beta implied by perfectly proportional allocation; (B) Coverage = % of high-need areas with any program presence; (C) Equity Gap Ratio = mean dosage in Q4 / Q1 of the CNI distribution.*

**Table S6. Descriptive Comparison of Implementing vs. Non-Implementing Regions**

| **Variable** | **N** | **Mean** | **SD** | **Min** | **Max** | **Group** |
| --- | --- | --- | --- | --- | --- | --- |
| Program dosage | 18,900 | 0.0551 | 0.1648 | 0.0000 | 0.9463 | Implementing |
| Relative CNI | 18,900 | 1.059 | 0.7675 | 0.0000 | 3.806 | Implementing |
| CNI score | 18,900 | 17.438 | 13.949 | 0.0000 | 79.404 | Implementing |
| Prop. foreign-born | 18,900 | 0.3054 | 0.2424 | 0.0000 | 1.000 | Implementing |
| Prop. unemployed | 18,900 | 0.1709 | 0.1769 | 0.0000 | 0.8851 | Implementing |
| Prop. low education | 18,900 | 0.0755 | 0.1244 | 0.0000 | 0.8320 | Implementing |
| Prop. single parent | 18,900 | 0.0935 | 0.0889 | 0.0000 | 0.5082 | Implementing |
| Program dosage | 46,913 | 0.0033 | 0.0412 | 0.0000 | 0.9245 | Non-Implementing |
| Relative CNI | 46,913 | 0.9761 | 0.6437 | 0.0000 | 3.853 | Non-Implementing |
| CNI score | 46,913 | 19.747 | 13.640 | 0.0000 | 79.116 | Non-Implementing |
| Prop. foreign-born | 46,913 | 0.2681 | 0.2264 | 0.0000 | 1.000 | Non-Implementing |
| Prop. unemployed | 46,913 | 0.2574 | 0.1696 | 0.0000 | 1.000 | Non-Implementing |
| Prop. low education | 46,913 | 0.0956 | 0.1205 | 0.0000 | 0.7737 | Non-Implementing |
| Prop. single parent | 46,913 | 0.1242 | 0.0766 | 0.0000 | 1.000 | Non-Implementing |

*Note: Means, standard deviations, and ranges for key variables across implementing and non-implementing regions. Implementing regions have higher proportions of foreign-born parents but lower unemployment rates.*

**Table S7. Sensitivity of Targeting Coefficient to Alternative Post-Rollout Threshold Definitions**

| **Threshold** | **N** | **CNI coefficient** | **SE** | **p-value** |
| --- | --- | --- | --- | --- |
| 0% (no restriction) | 18,900 | 0.076 | 0.004 | < 0.001 |
| 5% | 13,408 | 0.097 | 0.005 | < 0.001 |
| 10% | 11,571 | 0.103 | 0.005 | < 0.001 |
| 15% | 7,428 | 0.115 | 0.006 | < 0.001 |
| 20% | 6,517 | 0.111 | 0.006 | < 0.001 |
| 25% | 4,794 | 0.105 | 0.007 | < 0.001 |

Note: Each row re-estimates the main model (dosage ~ relative CNI, year FE, DeSO-clustered SE) using a different threshold to define the post-rollout start year for each region. The main analysis (N = 12,258) uses region-specific start years determined from the empirical rollout pattern; the strict 10% threshold yields a smaller sample because one region had coverage marginally below 10% in its first included year.

**Table S8. Two-Part Model Decomposition: Extensive Margin (Logit) and Intensive Margin (OLS)**

| **Model** | **N** | **CNI coef (base)** | **p-value (base)** | **CNI coef (expanded)** | **p-value (expanded)** |
| --- | --- | --- | --- | --- | --- |
| OLS (all observations) | 12,258 | 0.101 | < 0.001 | 0.150 | < 0.001 |
| Part 1: Logit (any dosage) | 12,258 | 1.342 | < 0.001 | -0.060 | 0.831 |
| Part 2: OLS (dosage \| > 0) | 3,298 | 0.058 | < 0.001 | 0.212 | < 0.001 |

Note: Two-part decomposition of the base and expanded OLS specifications. Part 1 is a logit model for any program presence (coefficients in log-odds); Part 2 is OLS conditional on non-zero dosage. Base models include only relative CNI and year fixed effects. Expanded models add proportion foreign-born, proportion unemployed, proportion low education, and proportion single parent. DeSO-clustered standard errors. The base model odds ratio for Part 1 is exp(1.342) = 3.83.

## Supplementary Figures


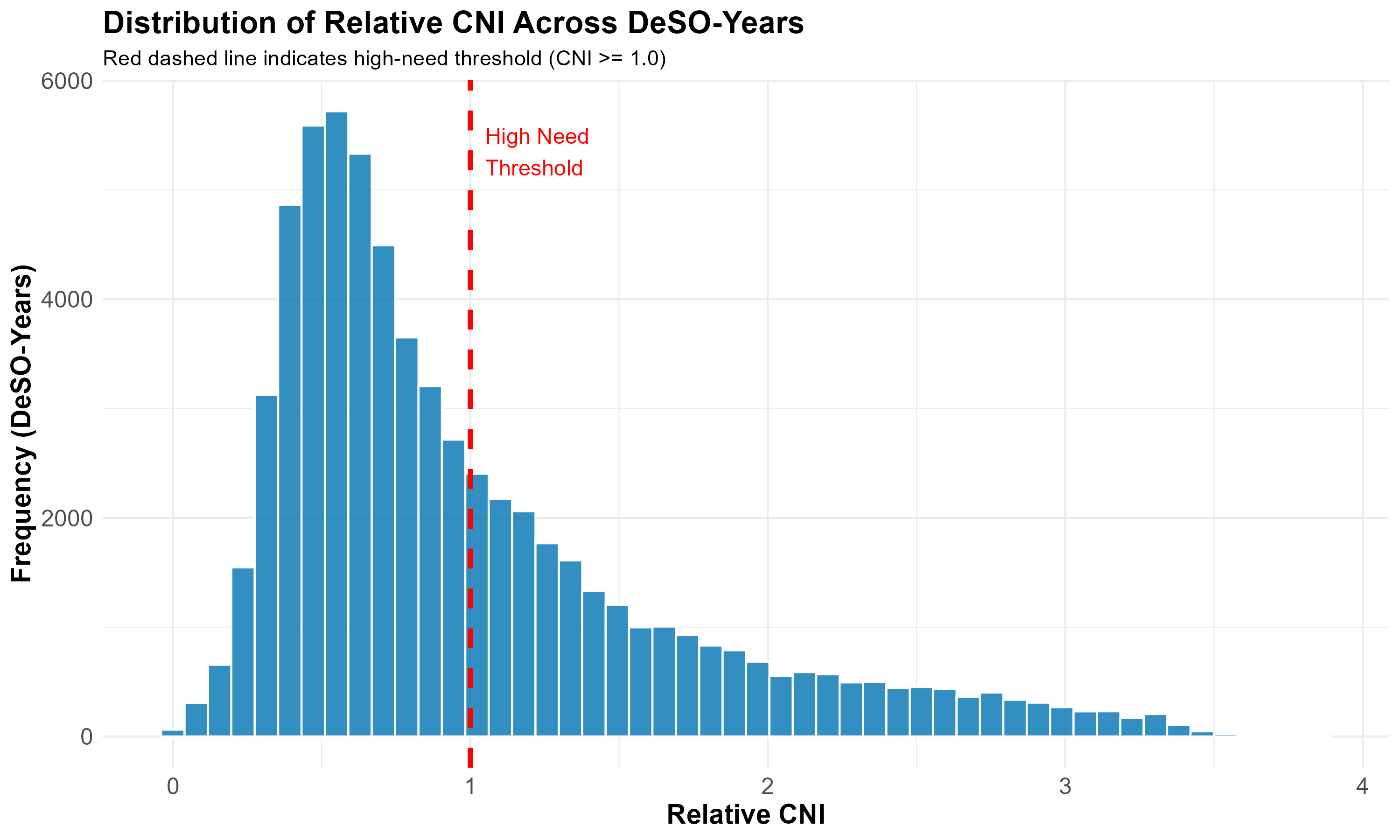


*Figure S1. Distribution of Relative CNI across DeSOs in implementing regions. The dashed line at 1.0 marks the national average; areas to the right are classified as 'high need.'*


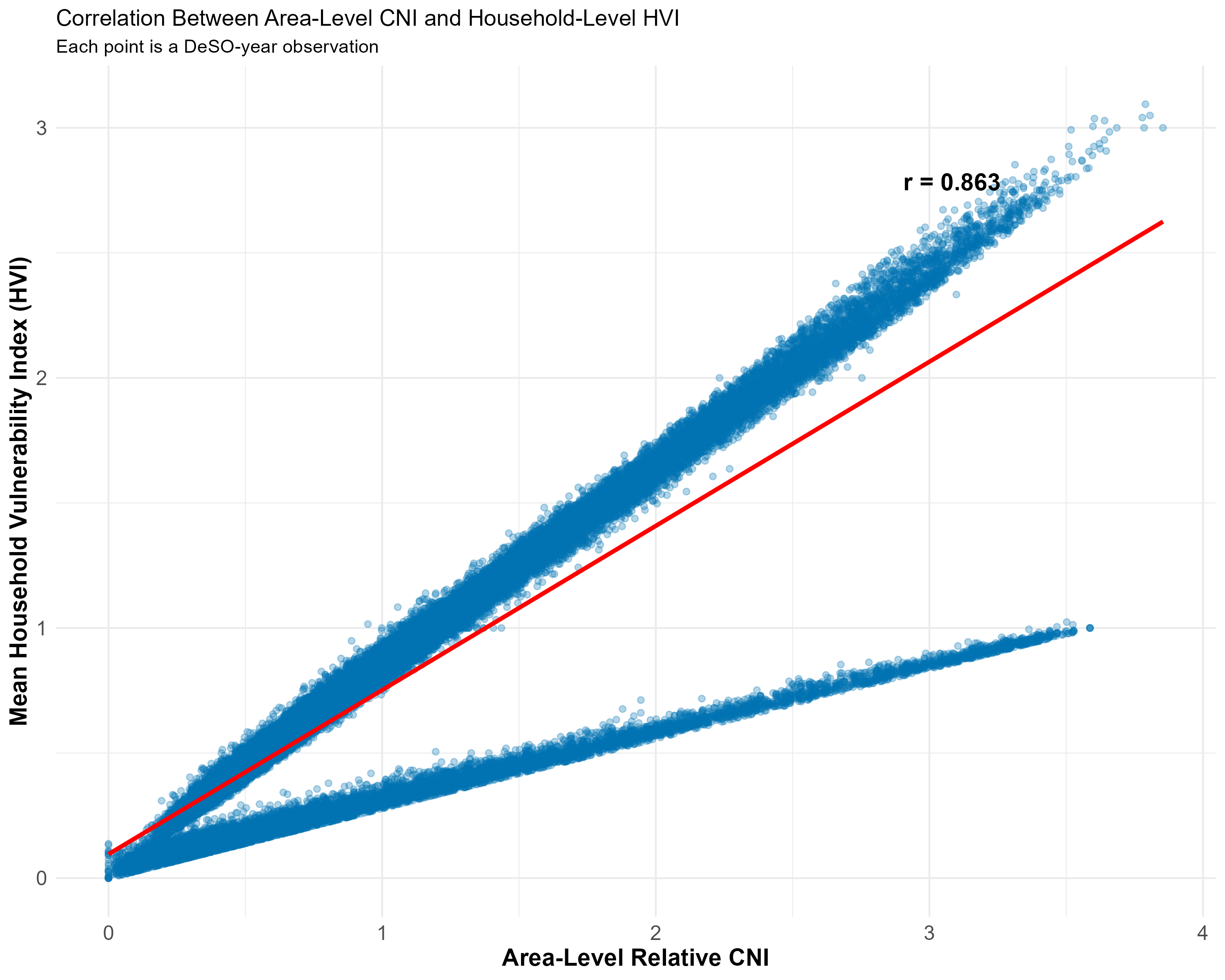


*Figure S2. Correlation between area-level Care Need Index (CNI) and aggregated Household Vulnerability Index (HVI). r = 0.86, indicating high but not perfect overlap between the two measures.*


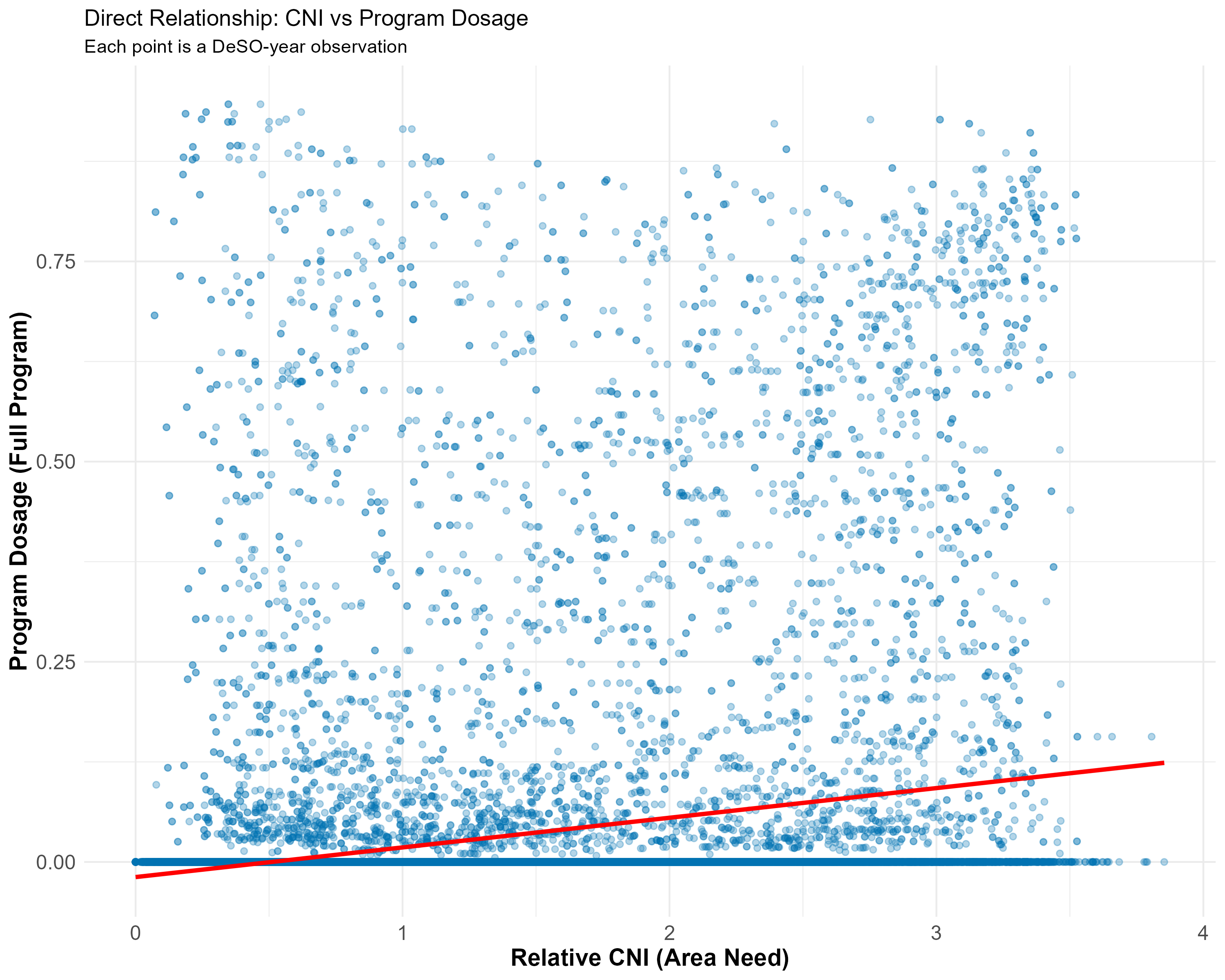


*Figure S3. Scatter plot of Relative CNI versus program dosage across DeSO-years. Note the high proportion of zero-dosage observations reflecting staged implementation.*


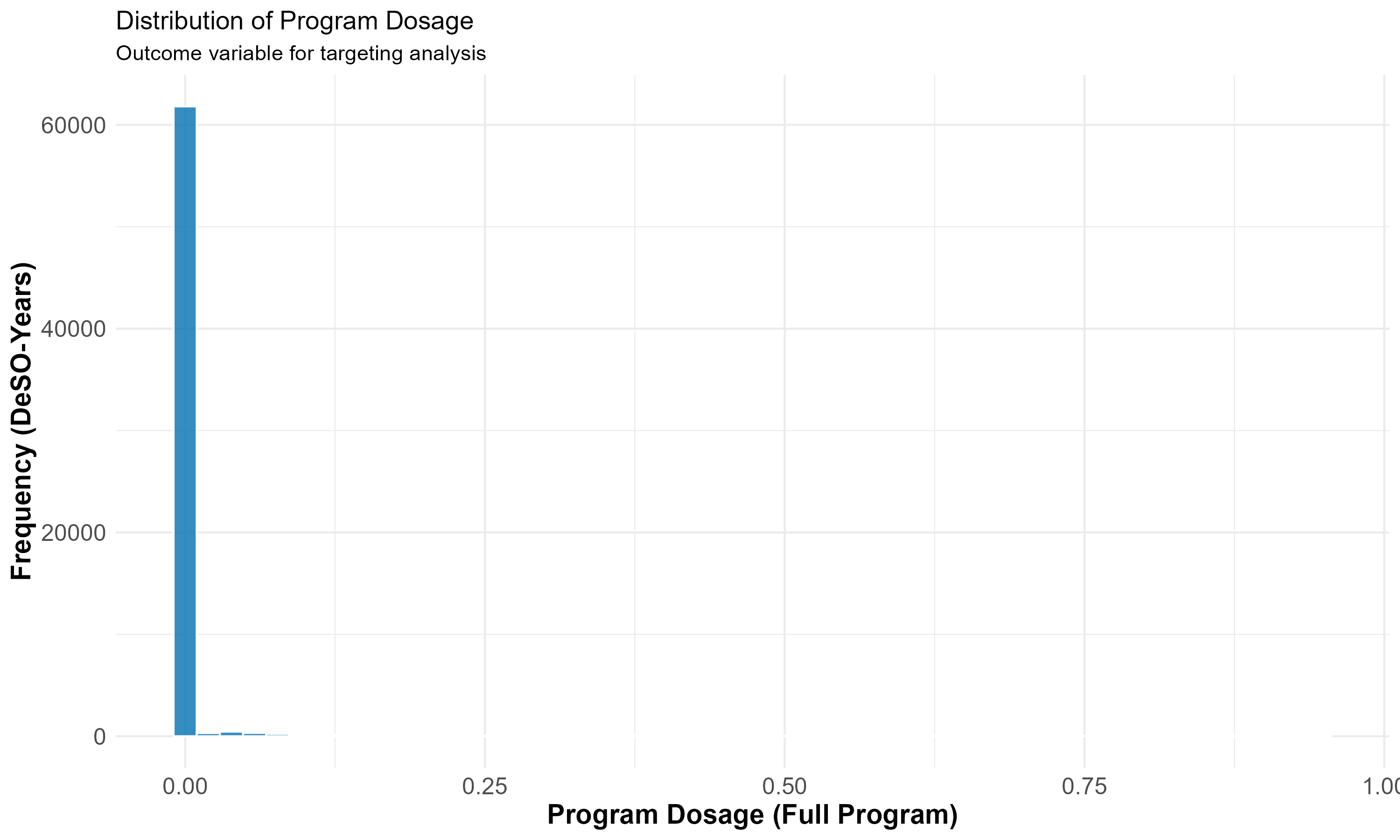


*Figure S4. Distribution of program dosage across DeSO-years. 73% of post-rollout DeSO-years have zero dosage. Among non-zero observations, mean dosage is 0.31.*


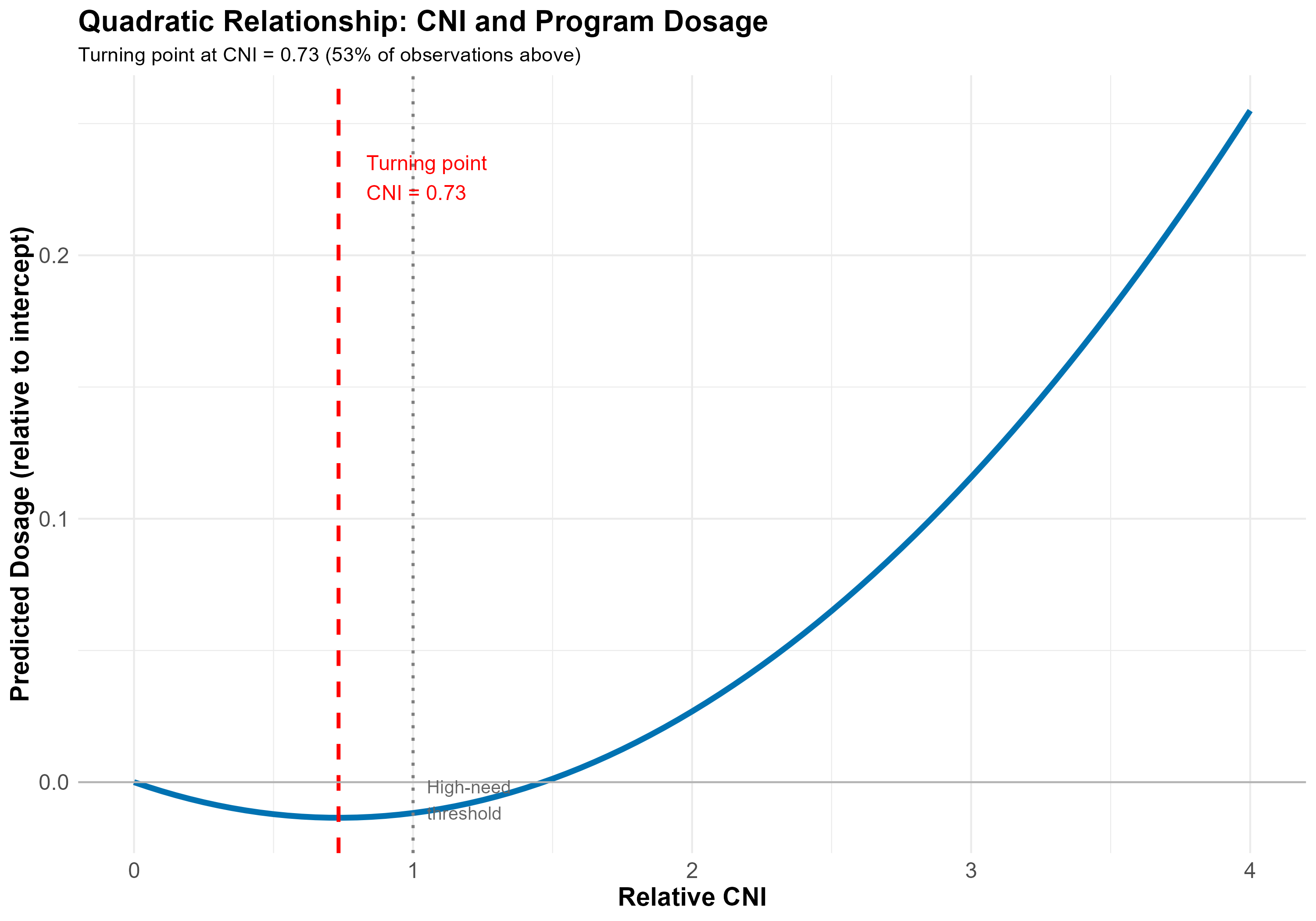


*Figure S5. Quadratic model: predicted dosage as a function of Relative CNI. The turning point at CNI = 0.49 marks where the marginal association between need and dosage shifts from negative to positive.*


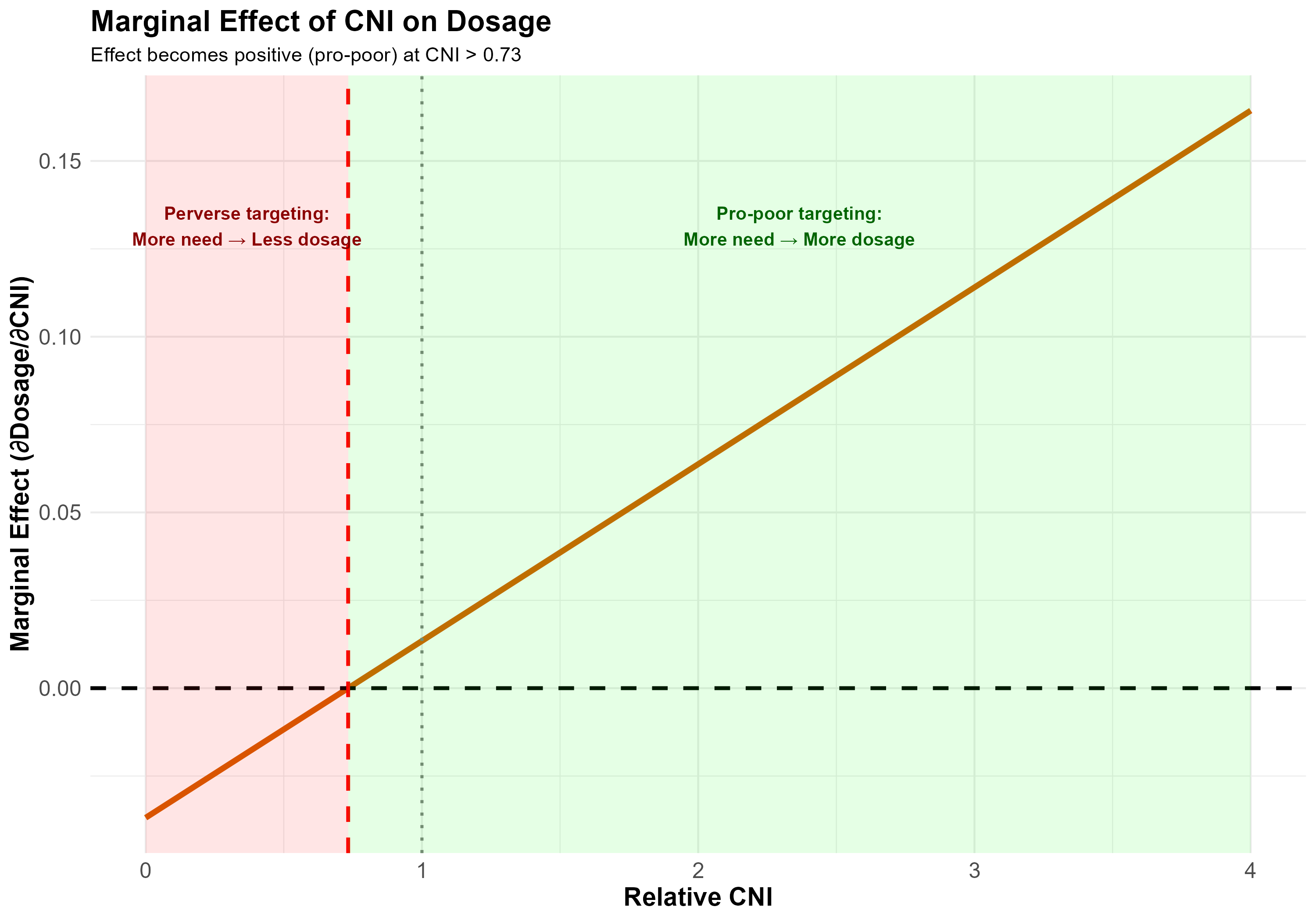


*Figure S6. Marginal effect of CNI on dosage across the need distribution. The effect becomes positive (pro-poor) above the turning point. 77% of post-rollout observations exceed this threshold.*


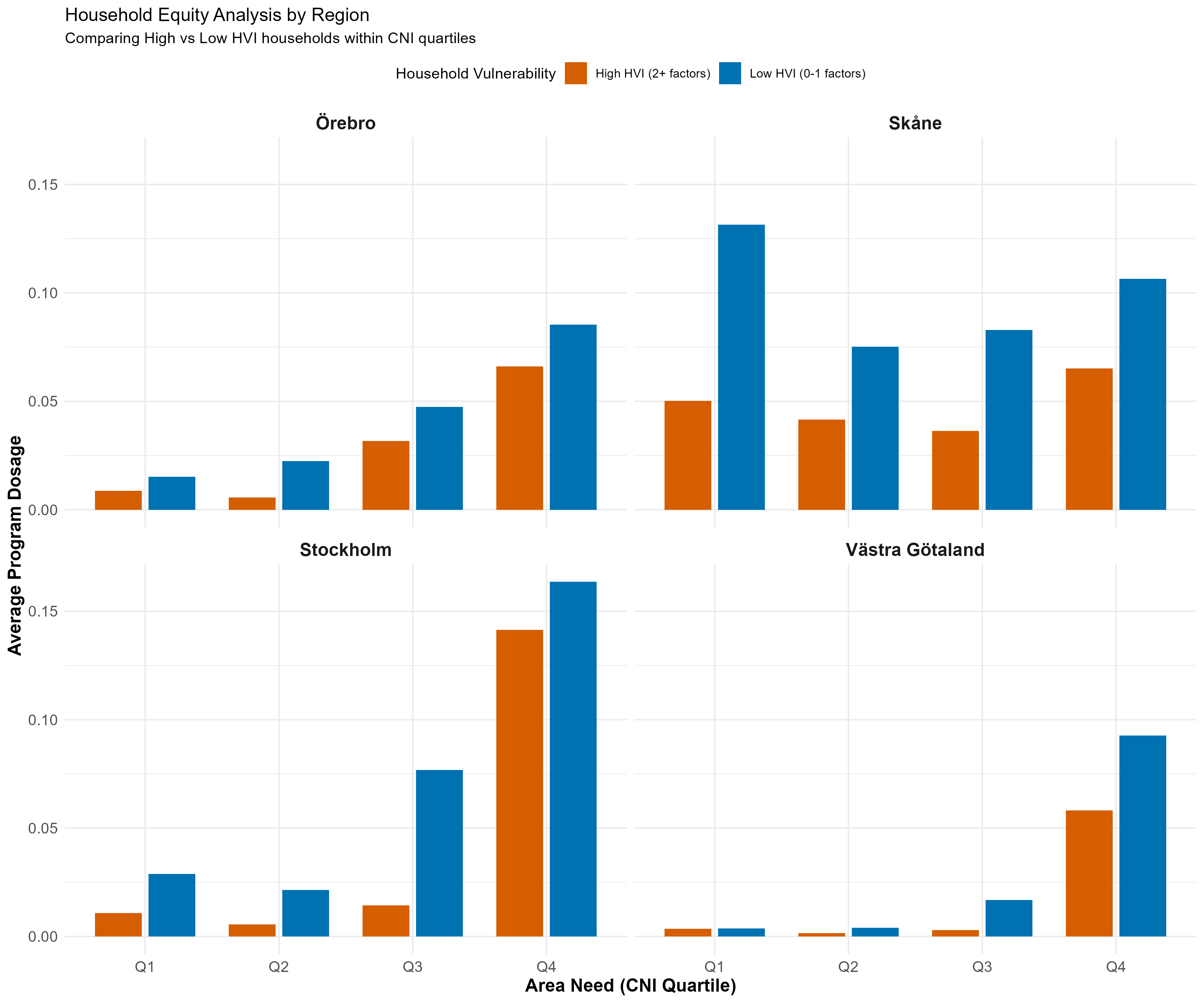


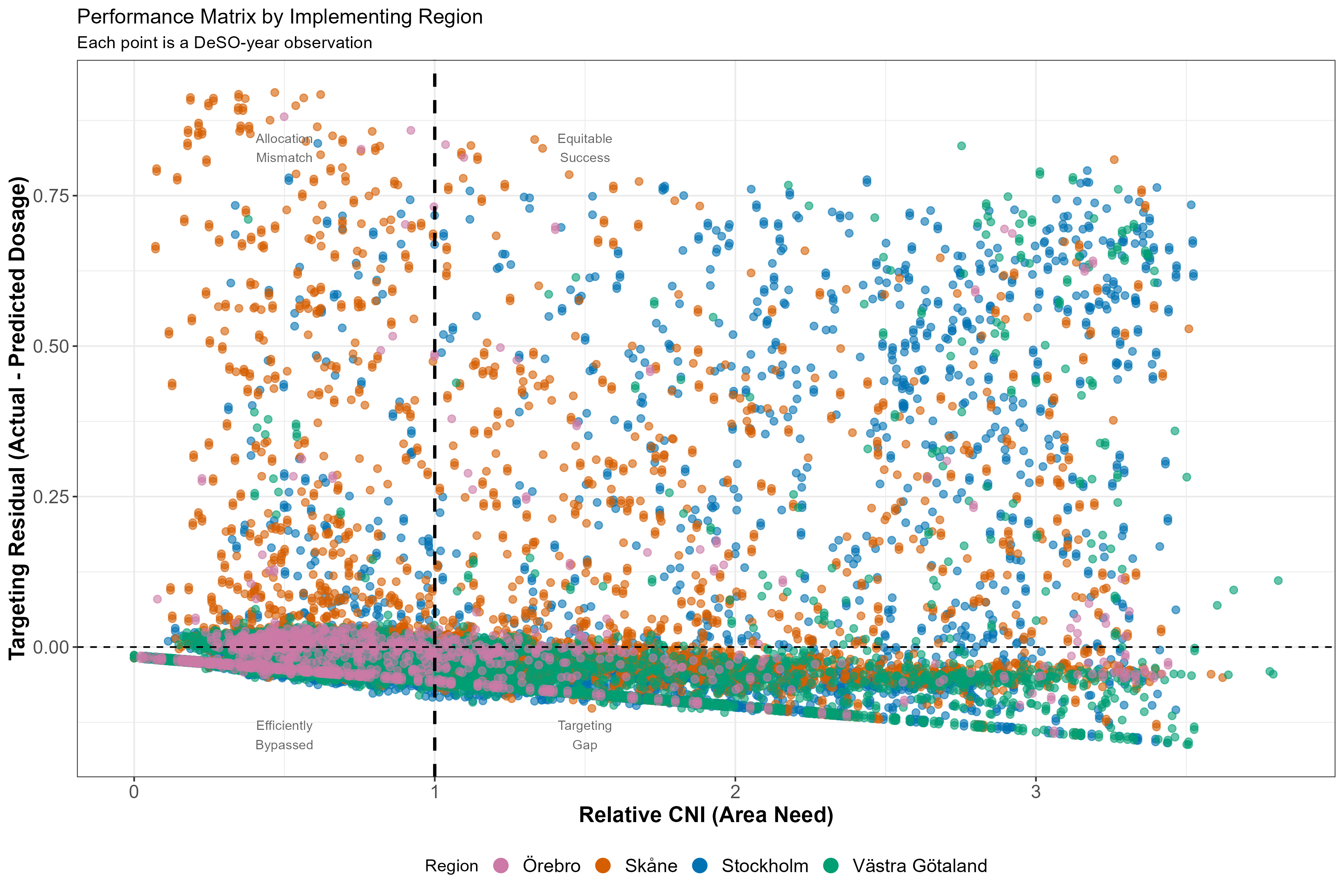

Supplement: Supplementary file 1 — Supplementary Material 1 [file 12939_2026_2867_MOESM1_ESM.docx]
